# Kaffir Doctors
*Papers relative to the State of the Kaffir Tribes. Parliamentary Report, 1856.


**Published:** 1856-07

**Authors:** 


### KAFFIR DOCTORS.*

In most cases of illness amongst the Kaffirs, the doctors profess to extract from the bodies of their patients substances said to have been introduced into their bodies through witchcraft. Wood, rags, cowdung, and reptiles are said to be thus extracted.

*Cases.* 1. Chronic deafness ; a boy ; twigs extracted from the ear. 2. Ulceration in the heel ; child ; name Refene ; had suffered for eighteen months ; friends consulted various doctors, who extracted sticks, thorns, and chips, but without success. Sacrifice was recommended as a last resource. 3. Case of ulceration of the nose ; a man ; suffered for two years. Doctors extracted various substances, but no cure. 4. Acute catarrh ; child ; several doctors consulted ; *they differed ;* some said a serpent had licked the child's head in the night ; others extracted a black substance from the forehead ; no success.

These cases afterwards came under Dr. Bindon of the 6th regiment, who found them all amenable to simple treatment. Dr. Bindon has had under his care in Kaffirland the following diseases : fevers, eye-diseases, chronic skin-diseases, consumption, asthma, inflammation of the bowels and lungs, lumbago, gonorrhœa, chronic ulcers, snake-bites, boils, ulceration of the tongue and mouth, deafness, heart-disease, epilepsy, catarrh, club-foot, amentia, and some cases of accident. The Kaffirs submit readily to European medical treatment.

* Papers relative to the State of the Kaffir Tribes. Parliamentary Report, 1856.

